# Physiologically Based Pharmacokinetic Modelling of Cabotegravir Microarray Patches in Rats and Humans

**DOI:** 10.3390/pharmaceutics15122709

**Published:** 2023-11-30

**Authors:** Hannah Kinvig, Rajith K. R. Rajoli, Henry Pertinez, Lalitkumar K. Vora, Fabiana Volpe-Zanutto, Ryan F. Donnelly, Steve Rannard, Charles Flexner, Marco Siccardi, Andrew Owen

**Affiliations:** 1 Department of Pharmacology and Therapeutics, Institute of Systems, Molecular and Integrative Biology, University of Liverpool, Liverpool L7 3NY, UK; hannah.kinvig@liverpool.ac.uk (H.K.);; 2 Centre of Excellence in Long-Acting Therapeutics (CELT), University of Liverpool, Liverpool L7 3NY, UK; 3 School of Pharmacy, Queen’s University Belfast, Medical Biology Centre, 97 Lisburn Road, Belfast BT9 7BL, UK; 4 Department of Chemistry, University of Liverpool, Liverpool L7 3NY, UK; 5 Department of Pharmacology and Molecular Sciences, Johns Hopkins School of Medicine, Baltimore, MD 21205, USA; flex@jhmi.edu

**Keywords:** cabotegravir, HIV, PrEP, long acting, PBPK, MAP

## Abstract

Microarray patches (MAPs) are currently under investigation as a self-administered, pain-free alternative used to achieve long-acting (LA) drug delivery. Cabotegravir is a potent antiretroviral that has demonstrated superior results over current pre-exposure prophylaxis (PrEP) regimens. This study aimed to apply physiologically based pharmacokinetic (PBPK) modelling to describe the pharmacokinetics of the dissolving bilayer MAP platform and predict the optimal dosing strategies for a once-weekly cabotegravir MAP. A mathematical description of a MAP was implemented into a PBPK model, and empirical models were utilised for parameter estimation. The intradermal PBPK model was verified against previously published in vivo rat data for intramuscular (IM) and MAP administration, and in vivo human data for the IM administration of LA cabotegravir. The verified model was utilised for the prediction of 300 mg, 150 mg and 75 mg once-weekly MAP administration in humans. Cabotegravir plasma concentrations >4 × protein-adjusted 90% inhibitory concentration (PA-IC_90_) (0.664 µg/mL) and >8 × PA-IC_90_ (1.33 µg/mL) were set as targets. The 75 mg, 150 mg and 300 mg once-weekly cabotegravir MAP regimens were predicted to sustain plasma concentrations >4 × PA-IC_90,_ while the 300 mg and 150 mg regimens achieved plasma concentrations >8 × PA-IC_90_. These data demonstrate the potential for a once-weekly cabotegravir MAP using practical patch sizes for humans and inform the further development of cabotegravir MAPs for HIV PrEP.

## 1. Introduction

Microarray patches (MAPs) offer a simple drug delivery method that provides pain-free self-administration and has the potential to provide long-acting (LA) treatment strategies across a range of indications. MAPs are considered minimally invasive and consist of multiple, micron-scale needle-like structures that penetrate the stratum corneum, viable epidermis, and dermis skin layers, delivering drugs through intradermal depot formation [1,2,3,4]. There are a wide variety of MAPs currently under investigation due to their advantages over alternative LA administration routes, such as intramuscular or subcutaneous injections. Relevant MAPs centre around solid, coated, hollow, dissolvable, biodegradable, and hydrogel-forming technologies [5], and could play a significant role in low- and middle-income countries (LMICs) because of their minimal manufacturing, storage and transportation costs, as well as their discreetness and ease-of-use [1,2,3,4].

MAPs are of particular interest in the prevention and treatment of human immunodeficiency virus (HIV), with patient surveys highlighting the desire for long-acting therapeutic strategies [6]. Approximately 74% of the 37.7 million people living with HIV received antiretroviral therapy in 2020 [7]. Current antiretroviral regimens contain two or three drugs and are predominantly orally administered on a daily basis [8,9,10,11]. Additionally, antiretrovirals are utilised as pre-exposure prophylaxis (PrEP) and post-exposure prophylaxis in HIV-negative individuals at high risk of HIV exposure [12]. A distinct advantage of LA strategies vs. daily oral regimens is their ability to overcome adherence issues, which remains a key challenge in HIV [6,13]. Recently, the first long-acting intramuscular (IM) injectable (containing cabotegravir and rilpivirine) was approved by several global regulatory agencies for once-monthly administration. Recent studies have suggested that the concentrations may also support IM administration once every 2 months, but this remains unproven and contentious [14,15,16].

Cabotegravir is an integrase strand transfer inhibitor that prevents the integration of the host chromosome and viral DNA by inhibiting the integrase enzyme responsible for covalent bond formation [11,17,18]. LA cabotegravir has also demonstrated its efficacy in HIV PrEP and has proven superior to daily oral tenofovir disoproxil fumarate and emtricitabine, particularly in at-risk populations such as cisgender men who have sex with men and transgender women [19,20,21,22,23,24]. A recently published study investigated a novel, bilayer MAP design containing LA cabotegravir in rats, and demonstrated a promising MAP design with high cabotegravir loading per cm^2^ [25]. A MAP intradermal PBPK model was previously reported, assessing the optimum characteristics for the MAP administration of cabotegravir and rilpivirine in HIV-negative humans, and described the movement of the drug from the MAP depot, through the layers of the skin and hair follicles, to its systemic circulation via the blood and lymphatic systems [26]. However, a specific empirical MAP design was not available at that time, and further modelling using published MAP characteristics is therefore warranted.

This study aimed to utilise in vivo rat data describing the cabotegravir pharmacokinetics resulting from dissolving bilayer MAPs for the qualification of a tailored MAP intradermal PBPK model. The qualified model was then used to predict cabotegravir pharmacokinetics in humans after once-weekly MAP administration, identifying the optimal doses for sustaining the target plasma concentrations for HIV PrEP.

## 2. Materials and Methods

A PBPK model was developed for the prediction of cabotegravir pharmacokinetics in Simbiology v5.8, a product of Matlab 2018a (MathWorks, Natick, MA, USA). Excluding the intradermal MAP compartments, drug distribution in the PBPK model was described using blood-flow-limited, first-order kinetics with well-stirred compartments that assumed instant distribution of the drug. Drug distribution in the intradermal MAP compartments was described using permeability-limited, first-order kinetics. Physicochemical, pharmacokinetic, in vitro, and in vivo data for cabotegravir were sourced from the literature or, if unavailable, were estimated using an empirical pharmacokinetic model in the R programming environment v4.0.3 (The R Foundation, Vienna, Austria) and via curve-fitting to observed data in the PBPK model. Where applicable, concentration time profile data were extracted from graphs using the Plot Digitizer Tool v4.5 (WebPlotDigitizer, Pacifica, CA, USA). For PBPK model predictions in rats, virtual cohorts of 100 rats were simulated. For PBPK model predictions in humans, virtual cohorts consisting of 50 male and 50 female patients aged 18–60 years were simulated.

### 2.1. Human and Rat Physiological Parameters

Human weight, height, body mass index and body surface area for virtual male and female patients aged 18–60 years were described using data from the National Center for Health Statistics [27,28,29]. Anthropometric equations were used to calculate human organ weight and alongside organ density and organ volume [28,30]. Blood flow rates in humans were determined as fractions of the total cardiac output, with the exception of dermal blood flow. Rat weight was described using mean ± standard deviation data reported for each cohort of rats (*n* = 6) used in the cabotegravir IM and MAP regimens utilised for model verification [25]. Rat organ volume [31], blood flow rates [31] and tissue composition [32] were defined as previously reported. The systemic circulation of cabotegravir for both humans and rats was calculated using differential equations for eliminating and non-eliminating organs, as previously reported [33]. Similarly, for both humans and rats the volume of distribution was calculated as described previously, with the zwitterionic olive oil:buffer partition coefficient (LogD^*^_vo:w_) equation being applied for cabotegravir [32].

### 2.2. MAP Intradermal PBPK Model

The physicochemical and pharmacokinetic input parameters for the simulation of cabotegravir in the MAP intradermal PBPK model are shown in Table 1. Due to the lack of available data, quantitative structure–property relationship (QSPR) equations were used to calculate permeability and partition coefficients for each skin layer, as previously described [26,34]. In order to optimise model parameters describing the nanoparticle release rate (K_NP_) in the microneedle depot and the rate of drug movement across the skin (K_SKIN_), plasma concentration data for cabotegravir was initially fitted with an empirical pharmacokinetic model in the R programming environment v4.0.3 (The R Foundation, Vienna, Austria). This fitting made use of the Pracma library and lsqnonlin function for non-linear regression [35]. The empirical model described one-compartment pharmacokinetic disposition for IM administration and two-compartment pharmacokinetic disposition for MAP administration, with first-order input from a dosing depot compartment representing the IM injection or MAP. Descriptions of the one-compartment and two-compartment empirical models can be found in Figures S1 and S2 and Equations (S1) and (S2), in the Supplementary Material, respectively. Parameters describing K_NP_ and K_SKIN_ were then implemented in the PBPK model. MAP K_NP_ and K_SKIN_ values verified in rats were utilised for human MAP predictions. Additionally, the apparent clearance for cabotegravir IM (CL/F_IM,rat_) and cabotegravir MAPs (CL/F_MAP,rat_) were estimated via curve-fitting to available in vivo rat data in the PBPK model. The apparent clearance for cabotegravir MAPs in humans (CL/F_MAP,human_) was estimated, allowing for a 20% bioavailability when compared to the apparent clearance of cabotegravir IM administration in humans (CL/F_IM,human_), as previously reported in rats [25].

**Figure 1:**
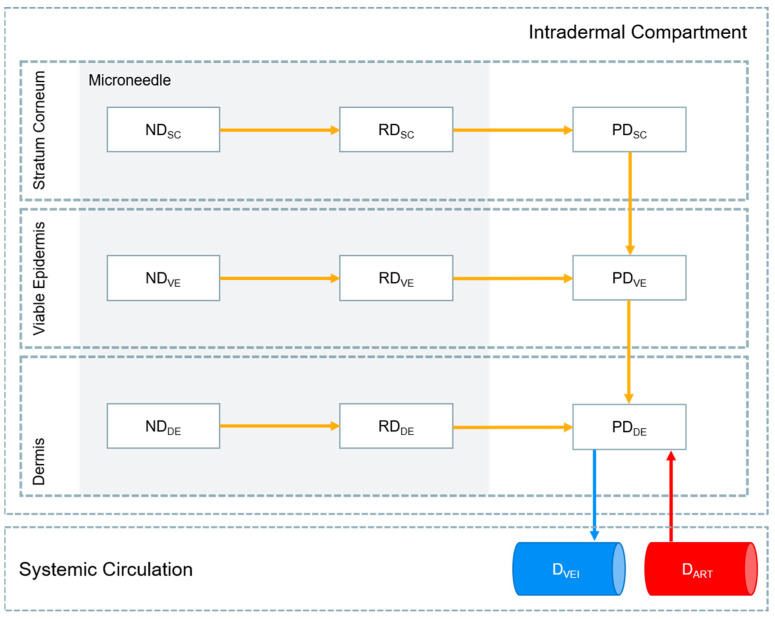
Schematic representation of the drug release pathway implemented in the MAP intradermal PBPK model. ND (nanoparticle drug), RD (released drug), PD (penetrating drug) and D (systemic drug) represent the drug in nanoparticle form in the microneedle, the drug released from its nanoparticle form in the microneedle, the released drug penetrating the layer of the skin and the drug in the systemic circulation, respectively. SC, VE, DE, VEI and ART represent the stratum corneum, viable epidermis, dermis, veins and arteries, respectively.

The MAP intradermal PBPK model was adapted from a previously reported model [26]. A schematic representation of the model is shown in Figure 1 and is described using differential Equations (1)–(9). Parameters utilised in Equations (1)–(9) are detailed in Table S1 in the Supplementary Material. The MAP drug dose was divided into the stratum corneum, viable epidermis and dermis layers of the skin based on the volume of the microneedle penetrating each layer. The thicknesses of the stratum corneum, viable epidermis and dermis implemented in the PBPK model were 18 µm, 32 µm and 2040 µm for rats and 17 µm, 47 µm and 2906 µm for humans, respectively [41]. As previously described in vivo, a dose loading of 5.86 mg/cm^2^ was implemented, with each microneedle baseplate (0.49 cm^2^) consisting of 16 × 16 right rectangular pyramid-shaped microneedles with a drug-free cuboidal base. The cuboidal base had a height of 250 µm with the right rectangular pyramid microneedle tip height equalling 600 µm. The width and length of each microneedle was 300 µm with a microneedle interspacing of 100 µm. A 97% insertion of the right rectangular pyramid microneedle tip was applied alongside no insertion of the cuboidal base [25]. Furthermore, the MAP intradermal PBPK model assumes that (1) only the drug released from the nanoparticle formulation can penetrate the layer of the skin and that this released drug can only travel in a unidirectional manner across different layers of the skin after it has left the microneedle depot; (2) the drug does not enter the hair follicles; (3) the drug enters and leaves the intradermal compartment via the bloodstream; and (4) K_NP_, permeability coefficients, partition coefficients and K_SKIN_ are constant.

The drug in the stratum corneum:

(1) $$\frac{{\mathrm{dND}}_{\mathrm{SC}}}{\mathrm{dt}}=-K_{\mathrm{NP}}\;\times \;{\mathrm{ND}}_{\mathrm{SC}}$$

(2) $$\frac{{\mathrm{dRD}}_{\mathrm{SC}}}{\mathrm{dt}}=K_{\mathrm{NP}}\;\times \;{\mathrm{ND}}_{\mathrm{SC}}-\;({\mathrm{PC}}_{\mathrm{SC}/W}\;\times \;{\mathrm{SA}}_{\mathrm{MN},\mathrm{SC}})\;\times \;\frac{{\mathrm{RD}}_{\mathrm{SC}}}{V_{\mathrm{MN},\mathrm{SC}}}$$

(3) $$\frac{{\mathrm{dPD}}_{\mathrm{SC}}}{\mathrm{dt}}=({\mathrm{PC}}_{\mathrm{SC}/W}\;\times \;{\mathrm{SA}}_{\mathrm{MN},\mathrm{SC}})\;\times \;\frac{{\mathrm{RD}}_{\mathrm{SC}}}{V_{\mathrm{MN},\mathrm{SC}}}-\left(\frac{1}{{\mathrm{PC}}_{\mathrm{SC}/\mathrm{VE}}}\;\times \;K_{\mathrm{SKIN}}\right)\;\times \;\frac{{\mathrm{PD}}_{\mathrm{SC}}}{V_{\mathrm{SC}}}$$

The drug in the viable epidermis:

(4) $$\frac{{\mathrm{dND}}_{\mathrm{VE}}}{\mathrm{dt}}=-K_{\mathrm{NP}}\;\times \;{\mathrm{ND}}_{\mathrm{VE}}$$

(5) $$\frac{{\mathrm{dRD}}_{\mathrm{VE}}}{\mathrm{dt}}=K_{\mathrm{NP}}\;\times \;{\mathrm{ND}}_{\mathrm{VE}}-({\mathrm{PC}}_{\mathrm{VE}/W}\;\times \;{\mathrm{SA}}_{\mathrm{MN},\mathrm{VE}})\;\times \;\frac{{\mathrm{RD}}_{\mathrm{VE}}}{V_{\mathrm{MN},\mathrm{VE}}}$$

(6) $$\begin{array}{c} \frac{{\mathrm{dPD}}_{\mathrm{VE}}}{\mathrm{dt}}=\left(\frac{1}{{\mathrm{PC}}_{\mathrm{SC}/\mathrm{VE}}}\;\times \;K_{\mathrm{SKIN}}\right)\;\times \;\frac{{\mathrm{PD}}_{\mathrm{SC}}}{V_{\mathrm{SC}}}+({\mathrm{PC}}_{\mathrm{VE}/W}\;\times \;{\mathrm{SA}}_{\mathrm{MN},\mathrm{VE}})\;\times \;\\ \frac{{\mathrm{RD}}_{\mathrm{VE}}}{V_{\mathrm{MN},\mathrm{VE}}}-\left(\frac{1}{{\mathrm{PC}}_{\mathrm{VE}/\mathrm{DE}}}\;\times \;K_{\mathrm{SKIN}}\right)\;\times \;\frac{{\mathrm{PD}}_{\mathrm{VE}}}{V_{\mathrm{VE}}} \end{array}$$

The drug in the dermis:

(7) $$\frac{{\mathrm{dND}}_{\mathrm{DE}}}{\mathrm{dt}}=-K_{\mathrm{NP}}\;\times \;{\mathrm{ND}}_{\mathrm{DE}}$$

(8) $$\frac{{\mathrm{dRD}}_{\mathrm{DE}}}{\mathrm{dt}}=K_{\mathrm{NP}}\;\times \;{\mathrm{ND}}_{\mathrm{DE}}-({\mathrm{PC}}_{\mathrm{DE}/W}\;\times \;{\mathrm{SA}}_{\mathrm{MN},\mathrm{DE}})\;\times \;\frac{{\mathrm{RD}}_{\mathrm{DE}}}{V_{\mathrm{MN},\mathrm{DE}}}$$

(9) $$\begin{array}{c} \frac{{\mathrm{dPD}}_{\mathrm{VE}}}{\mathrm{dt}}=\left(\frac{1}{{\mathrm{PC}}_{\mathrm{VE}/\mathrm{DE}}}\;\times \;K_{\mathrm{SKIN}}\right)\;\times \;\frac{{\mathrm{PD}}_{\mathrm{VE}}}{V_{\mathrm{VE}}}+({\mathrm{PC}}_{\mathrm{DE}/W}\;\times \;{\mathrm{SA}}_{\mathrm{MN},\mathrm{DE}})\;\times \;\\ \frac{{\mathrm{RD}}_{\mathrm{DE}}}{V_{\mathrm{MN},\mathrm{DE}}}+\;Q_{\mathrm{DE}}\;\times \;\frac{D_{\mathrm{ART}}}{V_{\mathrm{ART}}}-Q_{\mathrm{DE}}\;\times \;\frac{D_{\mathrm{VEI}}}{V_{\mathrm{DE}}/1000}\;\times \;\frac{R}{{\mathrm{TP}}_{\mathrm{SKIN}}} \end{array}$$

where ND, RD, PD and D represent the drug in its nanoparticle form in the microneedle, the drug released from its nanoparticle form in the microneedle, the released drug penetrating the layer of the skin and the drug in the systemic circulation, respectively. SC, VE, DE, VEI, ART and MN represent the stratum corneum, viable epidermis, dermis, veins, arteries and microneedle, respectively. SA and V are the surface area and volume, respectively. PC_SC/W_, PC_VE/W_ and PC_DE/W_ are the permeability coefficients between the stratum corneum and water, viable epidermis and water, and dermis and water, respectively. PC_SC/VE_ and PC_VE/DE_ are the partition coefficients between the stratum corneum and viable epidermis and between the viable epidermis and dermis. R and TP_SKIN_ are the blood-to-plasma ratio and tissue-to-plasma partition coefficient of the skin, respectively.

### 2.3. PBPK Model Verification

The MAP intradermal PBPK model was first verified against 2.5 mg/rat single-dose IM LA cabotegravir in vivo data in rats to validate the cabotegravir physicochemical parameters utilised in the model. The PBPK model was then verified against 11.72 mg/rat single-dose and once-weekly-dose LA cabotegravir MAP in vivo data in rats [25]. Following verification against rat data, human physiological characteristics were implemented into the MAP intradermal PBPK model and were verified against 800 mg single-dose IM LA cabotegravir in vivo data in HIV-negative humans [42]. The PBPK models were considered successfully verified if the ratio of predicted vs. observed pharmacokinetic values for the cabotegravir regimens in rats and humans were between 0.5–2, as per convention [26,43]. In addition, the absolute average fold error (AAFE) for the predicted vs. observed pharmacokinetic parameters and concentration time profiles were also calculated, as defined in Equation (10) [44]. AAFE values between 1 and 2 were considered successfully verified.

(10) $$\mathrm{AAFE}=10\left|\frac{1}{N}\;\Sigma \log\frac{\mathrm{Predicted}}{\mathrm{Observed}}\right|$$

### 2.4. Prediction of Cabotegravir MAP Pharmacokinetics in Humans

An initial MAP dosing regimen of once-weekly 300 mg cabotegravir with a dose loading of 5.86 mg/cm^2^ and patch size of 51.2 cm^2^ was simulated in humans based on previously reported in vivo studies in rats. MAP dosing regimens of 150 mg (25.6 cm^2^) and 75 mg (12.8 cm^2^) cabotegravir were also simulated to determine the smallest dose capable of sustaining effective plasma concentrations. K_NP_ and K_SKIN_ estimated in rats were applied in the human MAP intradermal PBPK model. A MAP bioavailability of 20% was applied in the prediction of cabotegravir pharmacokinetics in humans, as previously described [25]. A minimum cabotegravir plasma concentration of 4 × PA-IC_90_ (0.664 µg/mL) and 8 × PA-IC_90_ (1.33 µg/mL) of cabotegravir was targeted over a 6-month period [45].

## 3. Results

### 3.1. PBPK Model Verification

**Figure 2:**
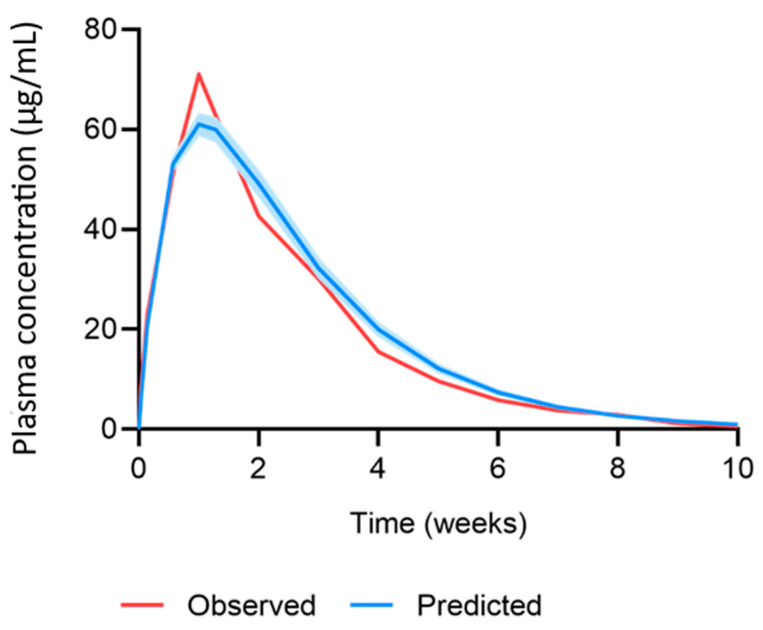
Observed vs. predicted concentration–time profile of cabotegravir after single-dose IM administration of 2.5 mg cabotegravir in rats. The red line represents the mean of the observed clinical data (µg/mL) [25]. The blue line and shaded area represent the mean plasma concentration ± standard deviation (µg/mL) of the predicted data.

**Figure 3:**
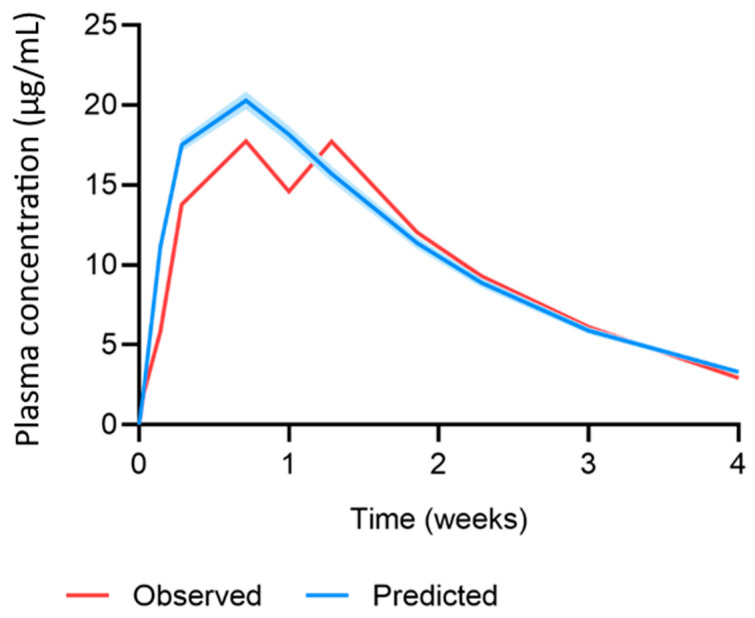
Observed vs. predicted concentration–time profile of cabotegravir after single-dose MAP administration of 11.72 mg cabotegravir in rats. The red line represents the mean of the observed clinical data (µg/mL) [25]. The blue line and shaded area represent the mean plasma concentration ± standard deviation (µg/mL) of the predicted data.

**Figure 4:**
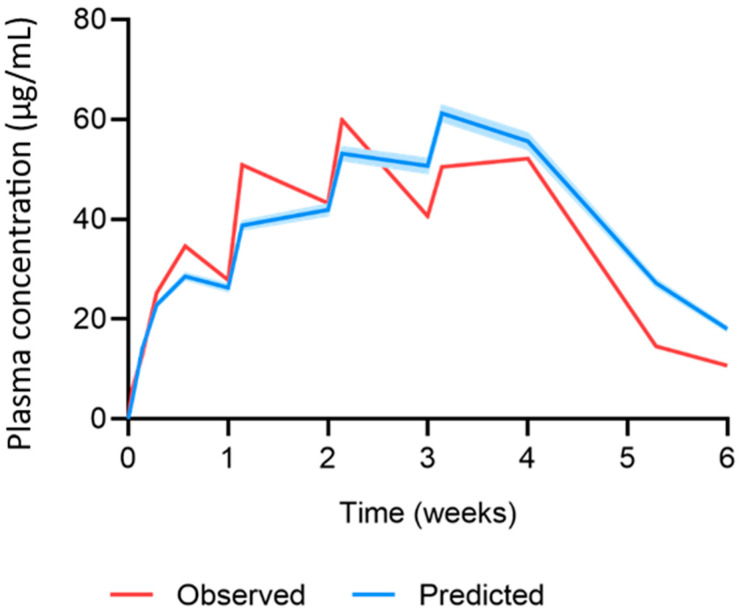
Observed vs. predicted concentration time profile of cabotegravir after once-weekly-dose MAP administration of 11.72 mg cabotegravir in rats. The red line represents the mean of the observed clinical data (µg/mL) [25]. The blue line and shaded area represent the mean plasma concentration ± standard deviation (µg/mL) of the predicted data.

**Figure 5:**
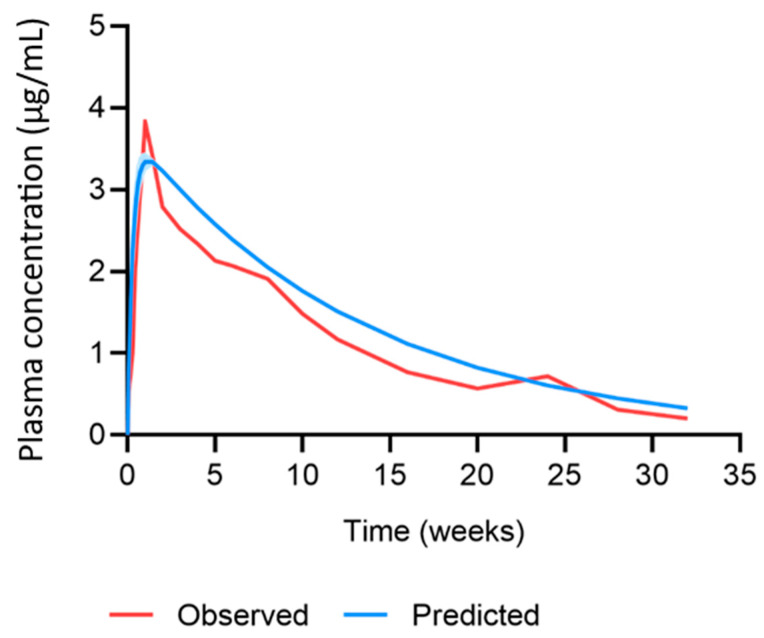
Observed vs. predicted concentration time profile of cabotegravir after single-dose IM administration of 800 mg cabotegravir in humans. The red line represents the mean of the observed clinical data (µg/mL) [42]. The blue line and shaded area represent the mean plasma concentration ± standard deviation (µg/mL) of the predicted data.

The MAP intradermal PBPK model was successfully verified according to the criteria by comparing the predicted pharmacokinetic parameters and concentration time profiles with the observed clinical data for IM and MAP administration. The AAFE and ratio verification results for the predicted cabotegravir pharmacokinetics from single-dose IM and MAP regimens in rats can be found in Table 2 and Table 3, respectively. The concentration–time profiles for single-dose IM, single-dose MAP and once-weekly-dose MAP in rats are shown in Figure 2, Figure 3 and Figure 4, respectively. The AAFE and ratio verification results for the predicted cabotegravir pharmacokinetics from single-dose IM administration in humans can be found in Table 4. The concentration–time profile for single-dose IM administration in humans is shown in Figure 5.

K_NP_ and K_SKIN_ were estimated and implemented in the PBPK models. The K_NP_ for IM and MAP administration in rats were 3 × 10^−3^ h^−1^ and 3.43 × 10^−3^ h^−1^, respectively. The K_SKIN_ for MAP administration was 1.73 × 10^−3^ cm^3^/h. CL/F_IM,rat_ and CL/F_MAP,rat_ were estimated as 0.6 L/h and 0.01 L/h, respectively. CL/F_MAP,human_ was calculated as 0.985 L/h, as previously described [25]. A correction factor was applied to the equations describing the volume of distribution, as per convention. For cabotegravir IM and MAP administration in rats, correction factors of 5 and 0.05 were applied. For cabotegravir IM and MAP administration in humans, a correction factor of 0.01 was applied.

### 3.2. Predicted Cabotegravir MAP Pharmacokinetics in Humans

**Figure 6:**
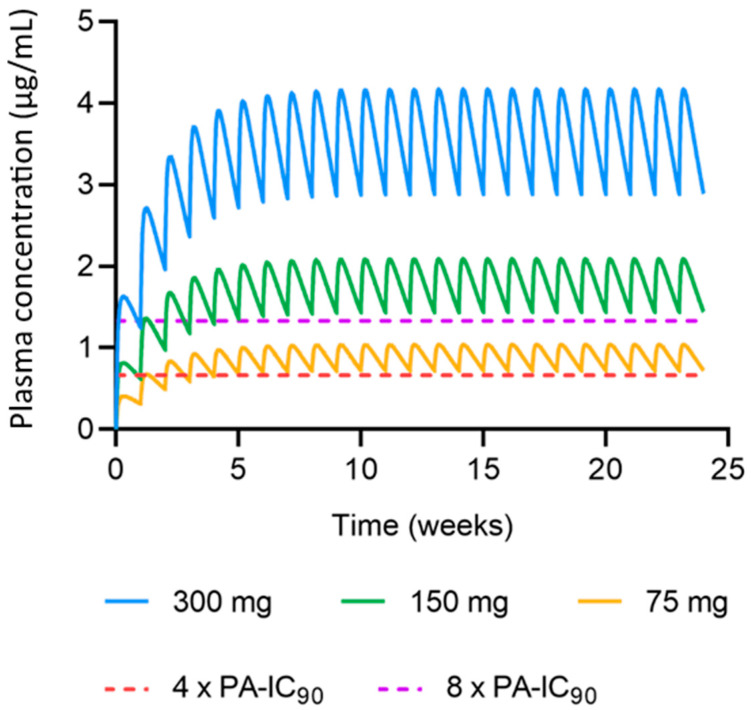
Predicted concentration–time profiles of cabotegravir following once-weekly MAP administration. The blue, green and yellow lines represent the mean plasma concentrations (µg/mL) of 300 mg (51.2 cm^2^), 150 mg (25.6 cm^2^) and 75 mg (12.8 cm^2^) for cabotegravir MAP administration, respectively. The red and purple dashed lines represent the minimum cabotegravir target concentrations of 4 × PA-IC_90_ (0.664 µg/mL) and 8 × PA-IC_90_ (1.33 µg/mL), respectively.

Human physiological characteristics were integrated into the MAP intradermal PBPK model to simulate cabotegravir pharmacokinetics resulting from the administration of MAPs in humans. A once-weekly cabotegravir MAP dose of 300 mg with a dose loading of 5.86 mg/cm^2^ and patch size of 51.2 cm^2^ was simulated with a nanoparticle release rate of 3.43 × 10^−3^ h^−1^ and MAP bioavailability of 20%, as previously described [25]. Additionally, once-weekly cabotegravir MAP doses of 150 mg (25.6 cm^2^) and 75 mg (12.8 cm^2^) were simulated. A minimum cabotegravir plasma concentration of 4 × PA-IC_90_ (0.664 µg/mL) and 8 × PA-IC_90_ (1.33 µg/mL) of cabotegravir were targeted [45]. The concentration–time profiles for all cabotegravir regimens can be found in Figure 6. The MAP intradermal PBPK model predicted that all cabotegravir MAP dosing regimens achieved cabotegravir concentrations greater than 4 × PA-IC_90_, while only the 150 mg and 300 mg cabotegravir MAP dosing regimens achieved concentrations of cabotegravir greater than 8 × PA-IC_90_. Specifically, cabotegravir MAP doses of 300 mg, 150 mg and 75 mg achieved a minimum concentration at steady state vs. 4 × PA-IC_90_ of 4.33, 2.16 and 1.08, respectively. Cabotegravir MAP doses of 300 mg and 150 mg achieved a minimum concentration at steady state vs. 8 × PA-IC_90_ of 2.16 and 1.08, respectively. However, while the 300 mg cabotegravir MAP sustained plasma concentrations above both minimum targets within 1 day of administration, the 150 mg and 75 mg cabotegravir MAPs did not. Plasma concentrations greater than 4 × PA-IC90 of cabotegravir were achieved 7 days and 28 days post MAP administration for the 150 mg and 75 mg doses, respectively, and plasma concentrations greater than 8 × PA-IC_90_ of cabotegravir were achieved 28 days post MAP administration for the 150 mg weekly dose.

## 4. Discussion

LA cabotegravir has recently been approved for long-acting IM administration in humans for HIV therapy (with LA rilpivirine) and PrEP (alone). Whilst current IM regimens offer once-monthly and bi-monthly administration [14,15,16], long-acting treatment options that are needle-free and self-administered are desirable. Several MAP technologies are currently under investigation and could offer a future alternative to oral and IM administration [1,2,3,4,5]. Unlike LA injectable formulations, MAPs are considered minimally invasive but are unlikely to provide an equivalent duration of exposure. Nonetheless, research has demonstrated an advantage to multiple treatment options, and MAPs may have a particular suitability for paediatric drug delivery, overcoming issues of tolerability and taste-masking in this oft under-studied patient group. In this study, in vivo rat data from a dissolving bilayer MAP device were utilised for the development and verification of a MAP intradermal PBPK model for the prediction of LA cabotegravir pharmacokinetics [25]. The PBPK model was used to predict the optimal MAP doses of cabotegravir for once-weekly administration in humans to evaluate cabotegravir MAPs as a potential candidate for PrEP.

The MAP intradermal PBPK model was verified against IM and MAP in vivo data in rats as well as IM clinical data in humans. Specifically, the simulated cabotegravir pharmacokinetics following IM administration in rats were within the conventional verification criteria. This dataset established the suitability of the parameters used to describe cabotegravir pharmacokinetics and rat physiology in the PBPK model. Generally, for IM administration in rats, the PBPK model tended to underpredict the C_max_ and overpredict the AUC, C_28_ and pharmacokinetic profile. The prediction of cabotegravir pharmacokinetics in rats following single-dose and multiple-dose MAP administration achieved the verification criteria, validating the mathematical description of the intradermal MAP in the PBPK model. When considering single-dose administration, all parameters were predicted well, with a tendency for overprediction. For multiple-dose MAP administration, the PBPK model underpredicted the C_max_ for the first dose, whilst the C_max_ at steady state was overpredicted. There was also a general trend in overpredicting the C_42_ and the pharmacokinetic profile. Interestingly, the C_max_ for the first dose was approximately 50% greater than the C_max_ found for the single-dose in vivo rat data, even though the MAP characteristics and dose were identical. This difference in pharmacokinetics creates challenges in PBPK model development and verification, as it is unclear as to whether the large variation is accurate or produced as a result of two differing experiments. Ideally, these studies would be replicated to confirm the current findings. Additionally, changes could be made to the in vivo protocol to verify further observations. For example, when analysing the predicted vs. observed pharmacokinetic profile in Figure 4, the observed C_max_ of the fourth dose is much lower than that of the second and third dose. This generates a discrepancy between the observed C_max_ and that of the predicted data, adversely affecting the ratio and AAFE verification results. The C_max_ observed for dose four may not have been accurately captured by the sampling protocol, whereby blood samples were taken prior to dose four, 24 h post dose and 7 days post dose. Considering the differences between doses, the C_max_ may have occurred in between these sampling timepoints.

Several parameters implemented in the PBPK model were estimated due to the lack of available data and must be taken into consideration when analysing the predicted PBPK model results. The K_NP_ for the IM and MAP administration of cabotegravir in rats as well as the K_SKIN_, were estimated using an empirical model. The estimated K_NP_ values were similar between both methods of administration, which is justifiable as the same formulations were used in the in vivo studies. A previous MAP intradermal PBPK model found that K_NP_ values between 7 × 10^−3^ and 9 × 10^−3^ h^−1^ would provide optimal cabotegravir plasma concentrations for a once-weekly 60 mg dose [26]. The estimated K_NP_ values in this study were approximately two to three times faster, thus highlighting an area in which the current MAP platform could be improved. Furthermore, the K_SKIN_ value was estimated to be 1.73 × 10^−3^ cm^3^/h^−1^, although additional in vivo and in vitro studies are required to determine if this estimation is accurate. However, this would be particularly challenging as several parameters were estimated in the mathematical description of the drug’s movement from the microneedle depot to systemic circulation, generating uncertainty in the reliability and accuracy of the predicted parameter values. This holds true for the K_NP_ as well as the QSPR calculated partition and permeability coefficients [26,34].

In addition to K_NP_ and K_SKIN_, CL/F_IM,rat_ and CL/F_MAP,rat_ were estimated in the PBPK model via curve-fitting to the observed clinical data. However, the CL/F estimated in the model for IM and MAP cabotegravir did not reflect the 20% bioavailability previously described [25]. This is the result of a nanoparticle dilution phenomenon reported in the in vivo rat studies. The 2.5 mg LA cabotegravir IM dose was simulated in the PBPK model due to its human-scaled dose being equivalent to the currently recommended 800 mg IM regimen. However, the 2.5 mg IM dose was diluted from the 10 mg dose stock during the in vivo study. Despite a difference in dose, the in vivo study reported similar C_max_ values for the 2.5 mg and 10 mg LA cabotegravir IM regimens. It is thought that this is due to an increase in absorption rate resulting from altered dissolution rates of the LA cabotegravir [25]. Due to a lack of data, the 20% bioavailability used to calculate CL/F_MAP,human_ from CL/F_IM,human_ remains justified, assuming that the LA cabotegravir simulated in humans is not produced via dilution. Lastly, a correction factor was applied to the volume of distribution to optimise model predictions for each regimen simulated in rats and humans, as per convention [46]. Nonetheless, the MAP intradermal PBPK model was verified against the criteria, demonstrating that the estimated parameters were capable of predicting the observed data.

The human physiological characteristics that were integrated into the MAP intradermal PBPK model and cabotegravir pharmacokinetics after IM administration in humans were predicted utilising available pharmacokinetic data. The PBPK model tended to overpredict the AUC, C_max_, C_W4_ and the concentration–time profile, albeit within the criteria. The PBPK model was applied for the prediction of cabotegravir pharmacokinetics in humans after MAP administration. A previously reported in vivo study in rats suggested that, based on current MAP technologies, the once-weekly MAP administration of cabotegravir following an 800 mg IM cabotegravir lead-in could sustain effective plasma concentrations with an appropriate MAP size [25]. Considering a bioavailability of 20%, this equates to a cabotegravir dose of 300 mg with a MAP size of ~50 cm^2^. Following these criteria, the PBPK model predicted that once-weekly MAP doses of cabotegravir between 300 mg and 75 mg (~13 cm^2^) could achieve plasma concentrations above the minimum target criteria of 4 × PA-IC_90_ and 8 × PA-IC_90_ of cabotegravir without an IM loading dose. Lower doses were predicted to take longer to achieve these criteria, with doses ≤150 mg reaching 4 × PA-IC_90_ and 8 × PA-IC_90_ of cabotegravir 7 days (75 mg) and 28 days (150 mg) after MAP administration, respectively. Whilst this data present promising results for cabotegravir as a candidate for once-weekly MAP administration, cabotegravir lacks the potency for once-monthly administration based on the high dose required and the impractical MAP sizes resulting from current technologies. Specifically, limited MAP bioavailability and drug loading present significant barriers to the successful implementation of MAP administration across antiretroviral drugs [1,2,3,4,5]. As mentioned, MAP bioavailability has been estimated as 20% when compared to IM administration in rats [25]. Whilst intravenous cabotegravir data would provide a more accurate representation of MAP bioavailability, it is clear that a MAP bioavailability >20% could greatly improve plasma exposure, reducing the MAP’s size and increasing MAP administration intervals across a range of antiretroviral drugs. Similarly, drug loading more than 3 mg/cm^2^ could produce similar results.

As highlighted, the MAP intradermal PBPK model in this study was developed from a previously published model [26]. Overall, the model described herein represents a simplified description of intradermal MAP pharmacokinetics, limiting the parameter estimation and model assumptions. There are four major differences in comparison to the previously reported model. Firstly, it was assumed that no drug enters the hair follicles. A previous study reported that the inclusion of hair follicles had a minimal impact on PBPK model performance [34]. Additionally, hair follicles have been reported to cover 0.1–20% of total skin, although it is unclear how accurate these values are, especially considering their large variation [34,47,48]. With a negligible role of hair follicles reported for drug transport, it was deemed an unnecessary complication for inclusion at this stage of model development [34,47]. Secondly, the equations used to describe drugs entering the lymphatic system produced little difference in PBPK model predictions [49]. To reduce model complexity and based on current knowledge gaps, the lymphatic system was excluded from the MAP intradermal PBPK model. Thirdly, no data are currently available detailing the rate of movement of the drug from MAPs across the skin layers. Therefore, by assuming that only the free drug can move unidirectionally across skin layers to the systemic circulation, less parameters require estimation, generating a model that favours simplicity. Lastly, in contrast to the right circular cone-shaped microneedle defined in a previous PBPK model [26], a right rectangular pyramid-shaped microneedle with a drug-free cuboidal base was implemented in the PBPK model described herein. Additionally, while acknowledging the physiological differences in skin thickness between males and females, our current analysis utilised mean values for both rats and humans due to data availability constraints.

Research initiatives investigating the above-mentioned assumptions, as well as additional mechanisms such as dermis vascularisation, could help fill knowledge gaps and improve MAP intradermal PBPK models’ prediction and reliability. However, future human pharmacokinetic data will be a prerequisite for identifying any inadequacies in the current model.

## 5. Conclusions

A MAP intradermal PBPK model was developed and verified utilising available rat and human pharmacokinetic data alongside empirical model-informed parameter estimation for the prediction of cabotegravir pharmacokinetics. Based on current MAP technologies, once-weekly cabotegravir doses of 75 mg, 150 mg and 300 mg were predicted to sustain effective plasma concentrations in humans with practical patch sizes between ~13 cm^2^ and ~50 cm^2^. Our study provides justification for further research into cabotegravir as a candidate for once-weekly MAP administration for HIV PrEP.

## Figures and Tables

**Table 1:** Physicochemical and pharmacokinetic input parameters for cabotegravir.

Parameter	Cabotegravir	Reference
Molecular Weight (g/mol)	405.358	[36]
HBD	2	[36]
Log P_O:W_	1.04	[36]
pK_a_	10.04, −0.7	[36]
Protein Binding (%)	99.8	[37]
PSA (Å2)	99.18	[36]
R	0.5	[37]
Bioavailability (%)	20	[25]
CL/F_IM,human_ (L/h)	0.197	[38]
K_np,IM,human_ (h^−1^)	4.54 × 10^−4^	[39]
PC_SC/W_	0.304 *	[26]
PC_VE/W_	0.106 **	[26]
PC_DE/W_	0.106 **	[26,40]
PC_SC/VE_	3.993 *	[26]
PC_VE/DE_	1 **	[40]

HBD—hydrogen bond donor, Log P_O:W_—partition coefficient between octanol and water, pK_a_—logarithmic value of the dissociation constant, PSA—polar surface area, R—blood-to-plasma ratio, CL/F_IM,human_—IM apparent clearance in humans, K_np,IM,human_—IM nanoparticle release rate in humans, PC_sc/w_—permeability coefficient between the stratum corneum and water, PC_VE/W_—permeability coefficient between the viable epidermis and water, PC_DE/W_—partition coefficient between dermis and water, PC_SC/VE_—partition coefficient between the stratum corneum and viable epidermis, PC_VE/DE_—partition coefficient between viable epidermis and dermis, *—calculated using QSPR equations as described previously [26], **—viable epidermis and dermis assumed to have similar compositions.

**Table 2:** Predicted vs. observed cabotegravir pharmacokinetics following single-dose IM administration of 2.5 mg cabotegravir in rats [25].

Parameter	Observed	Predicted	Ratio	AAFE
AUC_0–28_ (µg·day/mL)	1268.1 ± 290.3	1431.1 ± 67.9	1.13	1.13
C_max_ (µg/mL)	74.5 ± 11.6	61.1 ± 2.1	0.82	1.22
C_28_ (µg/mL)	14.5 ± 1.5	20 ± 1.3	1.38	1.38
Profile	-	-	1.10	-

Ratio data presented as predicted vs. observed. AAFE data were calculated as previously described. Observed clinical data presented as the mean ± standard deviation of six rats with a weight of 241 ± 16 mg [25]. Predicted data presented as the mean ± standard deviation of 100 simulated rats with a weight of 241 ± 16 mg. AUC_0–28_—area under the curve over 0–28 days, C_max_—maximum concentration, C_28_—concentration at day 28, Profile—concentration–time profile, -—not applicable, AAFE—absolute average fold error.

**Table 3:** Predicted vs. observed cabotegravir pharmacokinetics following single-dose and once-weekly-dose MAP administration of 11.72 mg cabotegravir in rats [25].

Dose (mg)	Parameter	Observed	Predicted	Ratio	AAFE
11.72 (single dose)	AUC_0–28_ (µg·day/mL)	291.1 ± 23.4	304.6 ± 7.6	1.05	1.05
	C_max_ (µg/mL)	18.1 ± 2.3	20.7 ± 0.5	1.15	1.15
	C_28_ (µg/mL)	3.1 ± 1.1	3.3 ± 0.09	1.06	1.06
	Profile	-	-	-	1.12
11.72 (once-weekly dose)	C_max-1_ (µg/mL)	35.1 ± 4.6	28.7 ± 0.7	0.82	1.22
	C_max-ss_ (µg/mL)	60.3 ± 10.7	67.4 ± 1.8	1.12	1.12
	C_42_ (µg/mL)	11.2 ± 2.2	17.9 ± 0.6	1.60	1.60
	Profile	-	-	-	1.04

Ratio data presented as predicted vs. observed. AAFE data were calculated as previously described. Observed clinical data presented as the mean ± standard deviation of six rats with a weight of 284 ± 10 for the single-dose study and 198 ± 8 mg for the once-weekly-dose study [25]. Predicted data are presented as the mean ± standard deviation of 100 simulated rats with weights corresponding to each study. AUC_0–28_—area under the curve over 0–28 days, C_max_—maximum concentration, C_max-1_—maximum concentration for the first dose, C_max-ss_—maximum concentration at steady state, C_28_—concentration at day 28, C_42_—concentration at day 42, Profile—concentration–time profile, -—not applicable, AAFE—absolute average fold error.

**Table 4:** Predicted vs. observed cabotegravir pharmacokinetics following single-dose IM administration of 800 mg cabotegravir in humans [25].

Parameter	Observed	Predicted	Ratio	AAFE
AUC_0–w4_ (µg·h/mL)	1497	1993.7 ± 48.3	1.33	1.33
AUC_0–w12_ (µg·h/mL)	3851	4792.7 ± 65	1.24	1.24
C_max_ (µg/mL)	3.3	3.4 ± 0.06	1.02	1.02
C_w4_ (µg/mL)	2	2.8 ± 0.02	1.39	1.39
Profile	-	-	-	1.23

Ratio data presented as predicted vs. observed. AAFE data were calculated as previously described. Observed clinical data are presented as the mean of six HIV-negative persons [42]. Predicted data are presented as the mean ± standard deviation of 100 simulated healthy male (50%) and female (50%) patients aged 18–60 years. AUC_0–w4_—area under the curve over 0–4 weeks, AUC_0-w12_—area under the curve over 0–12 weeks, C_max_—maximum concentration, C_w4_—concentration at week 4, Profile—concentration–time profile, -—not applicable, AAFE—absolute average fold error.

## Data Availability

Data are contained within the article and Supplementary Material.

## Supplementary Materials

The following supporting information can be downloaded at https://www.mdpi.com/article/10.3390/pharmaceutics15122709/s1, Figure S1: Schematic representation of the one-compartment empirical model describing the pharmacokinetic disposition of cabotegravir after intramuscular administration, Figure S2: Schematic representation of the two-compartment empirical model describing the pharmacokinetic disposition of cabotegravir after MAP administration, Table S1: MAP intradermal PBPK model parameters, Equation (S1): two-compartment empirical model, and Equation (S2): one-compartment empirical model. Reference [50] is cited in the Supplementary Materials.
