# Supplementary material for: Physiologically Based Pharmacokinetic Modelling of Cabotegravir Microarray Patches in Rats and Humans
Source: Pharmaceutics. 2023 Nov 30;15(12):2709. doi: 10.3390/pharmaceutics15122709 (PMC10747499; doi:10.3390/pharmaceutics15122709)
Supplement: Supplementary file 1 [file pharmaceutics-15-02709-s001.zip › pharmaceutics-2666226-supplementary.pdf]

# Physiologically Based Pharmacokinetic Modelling of Cabotegravir Microarray Patches in Rats and Humans

Hannah Kinvig [1,2], Rajith K.R. Rajoli [1,2], Henry Pertinez [1,2], Lalitkumar K. Vora [3], Fabiana Volpe-Zanutto [3], Ryan F. Donnelly [3], Steve Rannard [2,4], Charles Flexner [5], Marco Siccardi [1], Andrew Owen [1,2,*]

[1]Department of Pharmacology and Therapeutics, Institute of Systems, Molecular and Integrative Biology, University of Liverpool, Liverpool L7 3NY, UK

[2]Centre of Excellence in Long-Acting Therapeutics (CELT), University of Liverpool, Liverpool L7 3NY, UK

[3]School of Pharmacy, Queen's University Belfast, Medical Biology Centre, 97 Lisburn Road, Belfast BT9 7BL, UK.

[4]Department of Chemistry, University of Liverpool, Liverpool L7 3NY, UK

[5]Department of Pharmacology and Molecular Sciences, Johns Hopkins School of Medicine, Baltimore, MD 21205, USA.

*Correspondence: aowen@liverpool.ac.uk; Tel.: +44-(0)-151-794-8211

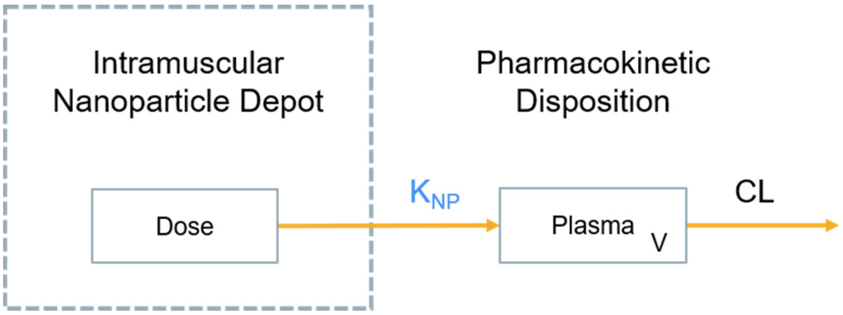


**Figure S1.** Schematic representation of the one-compartment empirical model describing the pharmacokinetic disposition of nanoformulated cabotegravir after intramuscular administration. Parameters highlighted in blue were implemented into the PBPK model. $K_{NP}$, V and CL represent the nanoparticle release rate, plasma compartment volume and clearance, respectively.

**Equation S1.** One-compartment empirical model.

$$\frac{dDose}{dt} = - K_{NP} \times Dose \tag{1}$$

$$\frac{dPlasma}{dt} = K_{NP} \times Dose/V - CL \times Plasma \tag{2}$$

Where $K_{NP}$, V and CL are the nanoparticle release rate, plasma compartment volume and clearance, respectively.

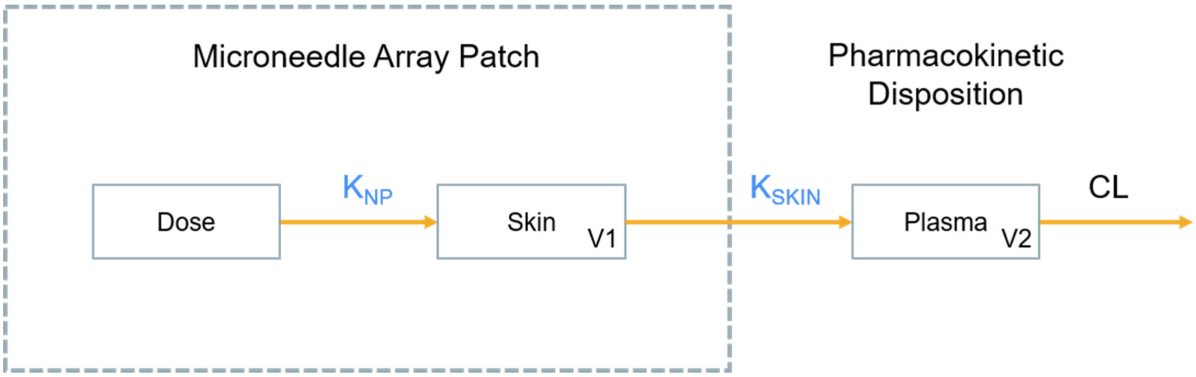


**Figure S2.** Schematic representation of the two-compartment empirical model describing the pharmacokinetic disposition of nanoformulated cabotegravir after microneedle array patch administration. Parameters highlighted in blue were implemented into the PBPK model. $K_{NP}$, $K_{SKIN}$, V1, V2 and CL represent the nanoparticle release rate, rate of drug movement in the skin, skin compartment volume, plasma compartment volume and clearance, respectively.

**Equation S2.** Two-compartment empirical model.

$$\frac{dDose}{dt} = - K_{NP} \times Dose \quad (3)$$

$$\frac{dSkin}{dt} = K_{NP} \times Dose/V1 - K_{SKIN} \times Skin/V1 \quad (4)$$

$$\frac{dPlasma}{dt} = K_{SKIN} \times Skin/V1 - CL \times Plasma/V2 \quad (5)$$

Where $K_{NP}$, V1, V2 and CL are the nanoparticle release rate, skin compartment volume, plasma compartment volume and clearance, respectively.

**Table S1.** MAP intradermal PBPK model parameters.

| Equation | Description |
|---|---|
| **Skin Characteristics** | |
| $V_{SC} = V_{tBP,SC} - V_{tMN,SC}$ | Volume of skin in the stratum corneum within the boundaries of the MAP |
| $V_{VE} = V_{tBP,VE} - V_{tMN,VE}$ | Volume of skin in the viable epidermis within the boundaries of the MAP |
| $V_{DE} = V_{tBP,DE} - V_{tMN,DE}$ | Volume of skin in the dermis within the boundaries of the MAP |
| $V_{BP,SC} = BP_W \times BP_L \times T_{SC}$ | Volume of the stratum corneum within the boundaries of one MAP baseplate |
| $V_{BP,VE} = BP_W \times BP_L \times T_{VE}$ | Volume of the viable epidermis within the boundaries of one MAP baseplate |
| $V_{BP,DE} = BP_W \times BP_L \times T_{DE}$ | Volume of the viable epidermis within the boundaries of one MAP baseplate |
| $V_{tBP,SC} = V_{BP,SC} \times BP_t$ | Volume of stratum corneum within the boundaries of total MAP baseplates |
| $V_{tBP,VE} = V_{BP,VE} \times BP_t$ | Volume of viable epidermis within the boundaries of total MAP baseplates |
| $V_{tBP,DE} = V_{BP,DE} \times BP_t$ | Volume of dermis within the boundaries of total MAP baseplates |
| $T_{SC}$ = 17 µM (human), $T_{SC}$ = 18 µM (rat) | Thickness of the *stratum corneum* [1] |
| $T_{VE}$ = 47 µM (human), $T_{SC}$ = 32 µM (rat) | Thickness of the viable epidermis [1] |
| $T_{DE}$ = 2906 µM (human), $T_{SC}$ = 2040 µM (rat) | Thickness of the dermis [1] |
| **MAP Characteristics** | |
| $BP_{MN} = 256$ | Number of microneedles per 16 ×16 baseplate [2] |
| $BP_t = \text{Dose}/BP_{LOADING}$ | Number of baseplates in MAP, based on dose |

| | |
|---|---|
| $BP_L = 0.7$ cm | Baseplate length [2] |
| $BP_W = 0.7$ cm | Baseplate width [2] |
| $MN_H = 600/10000$ | Microneedle height (cm) [2] |
| $MN_W = 300/10000$ | Microneedle width (cm) [2] |
| $MN_L = 300/10000$ | Microneedle length (cm) [2] |
| $MN_{H,SC} = T_{SC}$ | Height of a microneedle in the *stratum corneum* |
| $MN_{H,VE} = T_{VE}$ | Height of a microneedle in the viable epidermis |
| $MN_{H,DE} = MN_H – T_{SC} – T_{VE}$ | Height of a microneedle in the dermis, based on the remaining microneedle height after *stratum corneum* and viable epidermis penetration |
| $MN_{W,DE} = MN_W \times MN_{H,DE}/MN_H$ | Width of a microneedle in the dermis, based on the fraction of microneedle height |
| $MN_{L,DE} = MN_L \times MN_{H,DE}/MN_H$ | Length of a microneedle in the dermis, based on the fraction of microneedle height |
| $MN_{H,VEDE} = MN_{H,VE} + MN_{H,DE}$ | Height of a microneedle in the viable epidermis and dermis |
| $MN_{W,VEDE} = MN_W \times (MN_{H,DE} + MN_{H,VE})/MN_H$ | Width of a microneedle in the viable epidermis and dermis, based on the fraction of microneedle height |
| $MN_{L,VEDE} = MN_L \times (MN_{H,DE} + MN_{H,VE})/MN_H$ | Length of a microneedle in the viable epidermis and dermis, based on the fraction of microneedle height |
| $V_{MN} = (MN_H \times MN_W \times MN_L)/3$ | Volume of one microneedle |
| $V_{MN,SC} = V_{MN} – V_{MN,VEDE}$ | Volume of one microneedle in the *stratum corneum* |
| $V_{tMN,SC} = V_{MN,SC} \times BP_{MN} \times BP_t$ | Volume of total microneedles in the *stratum corneum* |
| $V_{MN,VE} = V_{MN,VEDE} – V_{MN,DE}$ | Volume of one microneedle in the viable epidermis |
| $V_{tMN,VE} = V_{MN,DE} \times BP_{MN} \times BP_t$ | Volume of total microneedles in the viable epidermis |
| $V_{MN,DE} = (MN_{H,DE} \times MN_{W,DE} \times MN_{L,DE})/3$ | Volume of one microneedle in the dermis |

| | |
|---|---|
| $V_{tMN,DE} = V_{MN,DE} \times BP_{MN} \times BP_{t}$ | Volume of total microneedles in the dermis |
| $V_{MN,VEDE} = (MN_{H,VEDE} \times MN_{W,VEDE} \times MN_{L,VEDE})/3$ | Volume of one microneedle in the viable epidermis and dermis |
| $SA_{MN} = MN_{H} \times MN_{W} + MN_{L}\sqrt{(MN_{W}/2)^2 + MN_{H}^2} + MN_{W}\sqrt{(MN_{L}/2)^2 + MN_{H}^2}$ | Surface area of one microneedle |
| $SA_{MN,SC} = SA_{MN} - SA_{MN,VEDE}$ | Surface area of one microneedle in the *stratum corneum* |
| $SA_{tMN,SC} = SA_{MN,SC} \times BP_{MN} \times BP_{t}$ | Surface area of total microneedles in the *stratum corneum* |
| $SA_{MN,VE} = SA_{MN,VEDE} - SA_{MN,DE}$ | Surface area of one microneedle in the viable epidermis |
| $SA_{tMN,VE} = SA_{MN,VE} \times BP_{MN} \times BP_{t}$ | Surface area of total microneedles in the viable epidermis |
| $SA_{MN,DE} = MN_{H,DE} \times MN_{W,DE} + MN_{L,DE}\sqrt{(MN_{W,DE}/2)^2 + MN_{H,DE}^2} + MN_{W,DE}\sqrt{(MN_{L,DE}/2)^2 + MN_{H,DE}^2}$ | Surface area of one microneedle in the dermis |
| $SA_{tMN,DE} = SA_{MN,DE} \times BP_{MN} \times BP_{t}$ | Surface area of total microneedles in the dermis |
| $SA_{MN,VEDE} = MN_{H,VEDE} \times MN_{W,VEDE} + MN_{L,VEDE}\sqrt{(MN_{W,VEDE}/2)^2 + MN_{H,VEDE}^2} + MN_{W,VEDE}\sqrt{(MN_{L,VEDE}/2)^2 + MN_{H,VEDE}^2}$ | Surface area of one microneedle in the viable epidermis and dermis |
| **Dermis Blood Flow** | |
| $Q_{DE} = V_{tBP,DE} \times (Q_{DERMIS} \times 60 \times 60/1000)$ | Blood flow rate in the dermis within the boundary of total MAP baseplates |
| $Q_{DERMIS} = 0.0022$ $cm^3$ blood/s/$cm^3$ skin | Blood flow rate in the dermis [3] |
| Microneedle characteristics based on a right rectangular pyramid shape, as previously described [2]. References presented in square brackets in the description column, where applicable. V - volume, t - total, BP - baseplate, MN - microneedle, SC - *stratum corneum,* VE - viable epidermis, DE - dermis, VEDE - viable epidermis and dermis, W - width, L - length, H - height, T - thickness, SA - surface area, Q – blood flow rate. | |

## References


1. Wei, J.C.J.; Edwards, G.A.; Martin, D.J.; Huang, H.; Crichton, M.L.; Kendall, M.A.F. Allometric scaling of skin thickness, elasticity, viscoelasticity to mass for micro-medical device translation: from mice, rats, rabbits, pigs to humans. *Sci Rep* **2017**, *7*, 15885, doi:https://doi.org/10.1038/s41598-017-15830-7.
2. Tekko, I.A.; Vora, L.K.; Volpe-Zanutto, F.; Moffatt, K.; Jarrahian, C.; McCarthy, H.O.; Donnelly, R.F. Novel Bilayer Microarray Patch-Assisted Long-Acting Micro-Depot Cabotegravir Intradermal Delivery for HIV Pre-Exposure Prophylaxis. *n/a*, 2106999, doi:https://doi.org/10.1002/adfm.202106999.
3. Ibrahim, R.; Nitsche, J.M.; Kasting, G.B. Dermal clearance model for epidermal bioavailability calculations. *J Pharm Sci* **2012**, *101*, 2094-2108, doi:https://doi.org/10.1002/jps.23106.
